# Modifier Effect in Silica-Supported FePO_4_ and Fe-Mo-O Catalysts for Propylene Glycol Oxidation

**DOI:** 10.3390/ma15051906

**Published:** 2022-03-04

**Authors:** Darya Y. Savenko, Mikhail A. Salaev, Valerii V. Dutov, Sergei A. Kulinich, Olga V. Vodyankina

**Affiliations:** 1 Laboratory of Catalytic Research, Tomsk State University, 36 Lenin Avenue, 634050 Tomsk, Russia; darya_ebert@mail.ru (D.Y.S.); mihan555@yandex.ru (M.A.S.); dutov_valeriy@mail.ru (V.V.D.); 2 Research Institute of Science & Technology, Tokai University, Hiratsuka 259-1292, Kanagawa, Japan; skulinich@tokai-u.jp; 3 School of Natural Sciences, Far Eastern Federal University, 690091 Vladivostok, Russia

**Keywords:** bio-regenerable sources, alcohol selective oxidation, iron molybdate catalysts, iron phosphate, bicarbonyl compounds, DFT

## Abstract

Currently, catalytic processing of biorenewable raw materials into valuable products attracts more and more attention. In the present work, silica-supported FePO_4_ and Fe-Mo-O catalysts are prepared, their phase composition, and catalytic properties are studied in the process of selective oxidation of propylene glycol into valuable mono- and bicarbonyl compounds, namely, hydroxyacetone and methylglyoxal. A comparative analysis of the main routes of propylene glycol adsorption with its subsequent oxidative conversion into carbonyl products is carried out. The DFT calculations show that in the presence of adsorbed oxygen atom, the introduction of the phosphate moiety to the Fe-containing site strengthens the alcohol adsorption on the catalyst surface with the formation of the 1,2-propanedioxy (–OCH(CH_3_)CH_2_O–) intermediate at the active site. The introduction of the molybdenum moiety to the Fe-containing site in the presence of the adsorbed oxygen atom is also energetically favorable, however, the interaction energy is found by 100 kJ/mol higher compared to the case with phosphate moiety that leads to an increase in the propylene glycol conversion while maintaining high selectivity towards C_3_ products. The catalytic properties of the synthesized iron-containing catalysts are experimentally compared with those of Ag/SiO_2_ sample. The synthesized FePO_4_/SiO_2_ and Fe-Mo-O/SiO_2_ catalysts are not inferior to the silver-containing catalyst and provide ~70% selectivity towards C_3_ products, while the main part of propylene glycol is converted into methylglyoxal in contrast to the Ag/SiO_2_ catalyst featuring the selective transformation of only the secondary C-OH group in the substrate molecule under the studied conditions with the formation of hydroxyacetone. Thus, supported Fe-Mo-O/SiO_2_ catalysts are promising for the selective oxidation of polyatomic alcohols under low-temperature conditions.

## 1. Introduction

Currently, the problem of the depletion of fossil natural resources with the accompanying complication of the global environmental situation is closely related to the increase in the demand for energy sources, fuels and chemicals. Therefore, the opportunity to use renewable raw materials, in particular biomass and biodiesel, to solve the challenges of modern energy as well as the development of new methods for biomass conversion into valuable organic intermediates used in various industries are among the trending research areas. Biomass is a renewable source of raw materials to produce fuels and a number of important organic compounds for chemical industries. Thus, polyatomic alcohols, in particular propylene glycol (PG) and glycerol, are the products of biomass conversion [1,2,3]. It is noteworthy that PG can also be obtained by the glycerol hydrogenolysis [4].

Experimental studies of the PG selective oxidation into carbonyl/carboxylic compounds (methylglyoxal, hydroxyacetone, etc.) are known in the literature for catalysts with supported metal nanoparticles, including Ag [5,6,7], Pt [8] and Pd [9,10,11,12]. The mechanism of PG conversion was theoretically studied over the Ag_4_ cluster [13]. As in the case of ethylene glycol oxidation [9,14,15], the PG adsorption on the surface of single crystal Ag (110) and Pd (111) coated with oxygen led to the breaking of O–H bonds with the formation of the intermediate 1,2-propanedioxy (–OCH(CH_3_)CH_2_O–, PDO) [5,6,9]. As the temperature increased, the PDO transformations yielded a number of products, including hydroxyacetone or acetol (CH_3_COCH_2_OH), lactaldehyde (CH_3_CH(OH)CHO), and methylglyoxal or pyruvaldehyde (CH_3_COCHO). Moreover, methylglyoxal and hydroxyacetone are of particular interest as the key intermediates with unique chemical properties due to the presence of two oxygen-containing groups. In Refs. [5,6], it was shown that, first of all, the C–H bond breaking on Ag (110) occurred at the secondary carbon atom of the PDO intermediate as evidenced by the low temperature of hydroxyacetone desorption. In Ref. [9], using the adsorption of ethylene glycol and PG on Pd (111) as an example it was shown that the intermediate compounds were strongly bound to the surface with Pd (111) in a planar orientation; as a result, glyoxal and methylglyoxal were desorbed in small amounts at temperatures above 250 K, however, they were primarily decarbonylated to form CO, H_2_ and methane. In Ref. [16], it was suggested that in the case of using the metal/bimetallic catalysts in the oxidation of polyatomic alcohols (e.g., PG and glycerol), the key role of the modifying metal was connected with controlling the reagent bond strength with the catalyst surface without a sharp change in the preferred geometry of the adsorbate, and such changes of binding energies can have a strong impact on the catalytic properties. Transition metals, such as Bi for Pd-Bi supported catalysts [12], are often used as a modifying metal added to those of the Pt or Au subgroups.

Catalysts based on transition metal compounds, such as Mo, Fe, V, are catalytically active in various oxidation processes making it possible to consider them as a promising alternative to those based on noble metals for selective conversion of alcohols and methane. In Ref. [17], the ethylene glycol reactions on the surface of the Mo (110) single crystal were considered in details by a complex of physical-chemical methods. It was shown that during the ethylene glycol adsorption on the surface of Mo (110), the intermediate compounds with the Mo-O bond were formed both in mono- (–OCH_2_CH_2_OH) and bidentate (–OCH_2_CH_2_O–) configurations. The authors found that bidentate species were more reactive due to their stability and greater propensity to form ethylene as compared to the potential transformations of the monodentate intermediates. High strength of the O–Mo bond caused the ethylene glycol deoxygenation to yield ethylene.

In Ref. [18], FePO_4_ catalysts were studied in direct oxidation of methane to methanol. Using Mössbauer spectroscopy coupled with XRD, the authors studied in details the phase transformations of the FePO_4_ catalysts in atmospheres of various oxidizers O_2_, H_2_O, and N_2_O. It was shown that the Fe_2_P_2_O_7_ phase dominated in the reduced catalyst. The use of H_2_O and N_2_O as oxidants in the selective conversion of methane to methanol promoted the formation of an active and selective mixed phase α-Fe_3_(P_2_O_7_)_2_ (Fe^3+^, Fe^2+^), while the formation of the less active phase β-Fe_3_(P_2_O_7_)_2_ was observed to a lesser extent, and the amount of the Fe_2_P_2_O_7_ phase decreased accordingly. It is worth noting that the Fe valence states can strongly affect the performance of Fe-based catalysts [19,20].

Due to the important industrial applications, the study of the surfaces of oxide iron-molybdenum catalysts for selective methanol oxidation into formaldehyde continues to attract the research attention [21,22]. In Ref. [22], the influence of the preparation method on the stability of the synthesized and industrial iron–molybdenum catalysts were studied. Thus, the catalysts prepared by hydrothermal method contained an excess of molybdenum represented by the metastable h-MoO_3_ phase, which, upon calcination, transformed into the thermodynamically stable α-MoO_3_ phase. The authors found that the crystal structure, crystal size and/or morphology of MoO_3_, taken in excess with respect to the iron molybdate, had a significant effect on the stability of the iron-molybdenum catalysts.

An approach involving the silver incorporation into the complex framework zirconium phosphates to prepare the catalysts active in the selective oxidation/dehydrogenation of ethanol to acetaldehyde was proposed in Ref. [23]. It was shown that the silver addition to complex zirconium phosphate catalysts led to a selectivity increase in the ethanol oxidation to acetaldehyde up to 74% at 330 °C at 93% ethanol conversion, significantly reducing the conversion along the dehydration route. The combination of redox and acid-base sites on the catalyst surface made it possible to control the main directions of transformations of polyatomic alcohols.

Thus, the studies aimed at finding the approaches and methods to assess the reactivity of different catalysts based on transition metal compounds, in particular Fe, using quantum-chemical calculations, are currently relevant to predict the surface properties of multicomponent catalysts. In previous works, when studying a series of supported FePO_4_/SiO_2_ [24] and Fe-Mo-O/SiO_2_ catalysts [25,26], it was shown that such catalysts were active and selective in the reaction of vapor-phase PG oxidation to methylglyoxal.

The present work is devoted to a comparison of the catalytic properties of FePO_4_/SiO_2_ and Fe-Mo-O/SiO_2_ catalysts using theoretical and experimental approaches. It has been shown that the theoretical estimation of the substrate–catalyst binding energy in comparison with the catalytic data can be a key to estimate the reactivity of intermediates depending on the chemical surrounding of the Fe sites.

## 2. Experimental Section

### 2.1. Catalyst Preparation

Silica gel of the KSKG brand (LLC “Salavat Catalyst Plant”, Salavat, Russia, S_BET_ = 300 m^2^/g), preliminarily dried in air at 110 °C for 15 h, was used as a support for the prepared catalysts. The amount of Fe in the FePO_4_/SiO_2_ and Fe-Mo-O/SiO_2_ samples calculated per iron (III) oxide was constant and amounted to 2.5 wt.% (Table 1).

Synthesis of the FePO_4_/SiO_2_ catalyst included two stages: impregnation of the silica gel fraction (0.25–0.5 mm) with a 0.2 M Fe(NO_3_)_3_ solution in terms of moisture capacity and subsequent impregnation with a (NH_4_)_3_PO_4_ solution of the appropriate concentration. The amount of the introduced P corresponded to the molar ratio P/Fe = 2.4 (Table 1). The prepared catalyst was dried at 110 °C and calcined in air at 600 °C for 8 h.

The Fe-Mo-O/SiO_2_ iron-molybdenum catalyst was prepared by co-impregnation with a citric acid solution containing both components, ammonium heptamolybdate ((NH_4_)_3_Mo_7_O_24_∙4H_2_O) and iron (III) nitrate in the appropriate concentrations based on Mo/Fe = 2 (mol.). The synthesized catalyst was dried at 120 °C and calcined at 550 °C in an air stream.

The Ag-containing sample with a silver content of 5 wt.% was used as a reference sample. The Ag/SiO_2_ catalyst was synthesized by impregnating the moisture capacity with an AgNO_3_ solution of the appropriate concentration [27]. Then, the sample was dried at 70 °C for 12 h, then the deposited Ag precursor was subjected to high-temperature treatment in an air flow at 500 °C followed by reduction at 200 °C in the H_2_/Ar flow. The specific surface area of the synthesized Ag/SiO_2_ sample was 170 m^2^/g.

Individual Fe_2_O_3_/SiO_2_ oxide catalyst was prepared as a reference sample through the wetness impregnation. The amount of supported component in the model sample was 10 wt.%. The resulting sample was dried at 110 °C and calcined at 500 °C. The specific surface area of the synthesized Fe_2_O_3_/SiO_2_ sample was 268 m^2^/g.

### 2.2. Catalyst Characterization

The phase composition of the samples was studied on the Miniflex 600 diffractometer (Rigaku, Japan) with a Cu anode in the range of 2θ = 10°–80° at a scanning rate of 2°/min. The phase composition was identified using the PDF-2 database and the full profile analysis program POWDER CELL 2.4. Elemental analysis of the catalysts was carried out by X-ray fluorescence analysis (XRF) using the X-ray fluorescence-wave dispersive spectrometer (XRF-1800, Shimadzu, Kyoto, Japan). The source was the X-ray tube with an Rh anode, a voltage was 40 kV, a current was 95 mA, and a diaphragm was 10 mm.

The textural characteristics of the synthesized samples were studied using the method of low-temperature nitrogen adsorption at 196 °C on the TriStar II 3020 analyzer (Micromeritics, Norcross, GA, USA). All samples were subjected to degassing (10^−2^ Torr) at 200 °C for 2 h prior to experiments. The specific surface was determined by the BET method. The pore size distribution was calculated from the desorption branch of the adsorption-desorption isotherm by the Barrett-Joyner-Halend (BJH) method.

### 2.3. Computational Details

The theoretical interpretation of the vapor-phase PG oxidation to methylglyoxal on FePO_4_/SiO_2_ and Fe-Mo-O/SiO_2_ catalysts was carried out by density functional theory (DFT) method using the Gaussian’09 software package installed on the SKIF “Cyberia” supercomputer of Tomsk State University [28]. The DFT calculations were carried out using the B3LYP functional. Modeling of the interactions of reagents (PG, O_2_), intermediates and products obtained during the PG conversion was carried out using the DGDZVP basis set (Geometries of substrates are represented in the Supplementary Materials (Figure S1)). To provide the effectiveness of time and computer costs, FePO_4_H and Fe_2_(MoO_4_)_3_ were taken as models of active sites for iron-phosphate and iron-molybdate catalysts, respectively. A hydrogen atom was added to FePO_4_ moiety to ensure conversion. The geometries of the main substrates considered, as well as DFT-optimized cartesian coordinates for the main FePO_4_- and Fe_2_(MoO_4_)_3_-based models, are represented in the Supplementary Materials (Figures S2 and S3).

The interactions of the main process components were modeled in accordance with the procedure described in Ref. [13] as well as on the basis of the reactions presented in Refs. [5,6] that occurred during the PG oxidation to methylglyoxal. The geometries of all structures obtained were fully optimized. In all cases, the stationary point nature was verified by calculating the vibrational frequencies. Most of the optimized structures were in global energy minimum and featured only real frequencies. The absence of imaginary vibrational frequencies confirmed the stationary nature of the structures. When there were imaginary frequencies in the structure, the IRC calculations were carried out to determine the transition state. The calculated thermodynamic parameters of the molecules were corrected for zero-point vibrational energy (ZPVE) and brought to normal conditions (298.15 K, 1 atm) using the thermal corrections to enthalpy and free energy. The interaction energy of the gas-phase molecule/intermediate with the active site of the catalyst (FePO_4_, Fe_2_(MoO_4_)_3_) was determined as the difference between the total energy of the substrate/active site system and the sum of the energies of the isolated substrate and the active site of the catalyst. In the case of oxygen-containing systems, the interaction energies were determined as the difference between the total energy of the oxygen-containing active site/substrate system and the sum of the energies of the isolated oxygen-containing active site and substrate.

### 2.4. Testing of Catalytic Activity

The catalytic properties of the synthesized catalysts were studied in the vapour-phase PG oxidation to methylglyoxal at a temperature of 350 °C (composition of the incoming mixture: 3 vol.% C_3_H_6_(OH)_2_, 3.7 vol.% O_2_, 63.3 vol.% N_2_, 30 vol.% H_2_O). A sample weighing of 0.15 g was loaded into a quartz reactor with a fraction of 0.25–0.5 mm; the reaction mixture was fed at a rate of 1.5 L/min. The reaction products were analyzed by gas chromatography (GC) and high-performance liquid chromatography (HPLC). The PG conversion for iron-molybdenum catalysts was calculated according to formula (1):

(1) $$X=\frac{\sum_{i}an_{i}}{3n{\left(PG\right)}_{inlet}}\times 100\%$$

where *n_i_* was the number of moles of the *i*th product (*i*—methylgloxal (MeGO), hydroxyacetone (HA), formaldehyde (FD), CO and CO_2_); *a* was the number of carbon atoms in the *i*thproduct. The selectivities towards the reaction products was calculated in accordance with the presented formulas (2)–(5):

(2) $$S_{MeGO}=\frac{n_{MeGO}}{\sum_{i}an_{i}}\times 100\%;\;$$

(3) $$S_{HA}=\frac{n_{HA}}{\sum_{i}an_{i}}\times 100\%;\;$$

(4) $$S_{FD}=\frac{n_{FD}}{3\sum_{i}an_{i}}\times 100\%;\;$$

(5) $$S_{CO_{x}}=\frac{n_{CO_{x}}}{3\sum_{i}an_{i}}\times 100\%.\;$$

## 3. Results and Discussion

Table 1 lists the designations of the synthesized catalysts, the amounts of deposited components according to the XRF results and the specific surface area. Both iron-containing catalysts are characterized by similar Fe content (per iron (III) oxide) as well as a close molar ratio [P or Mo]/Fe.

**Figure 1:**
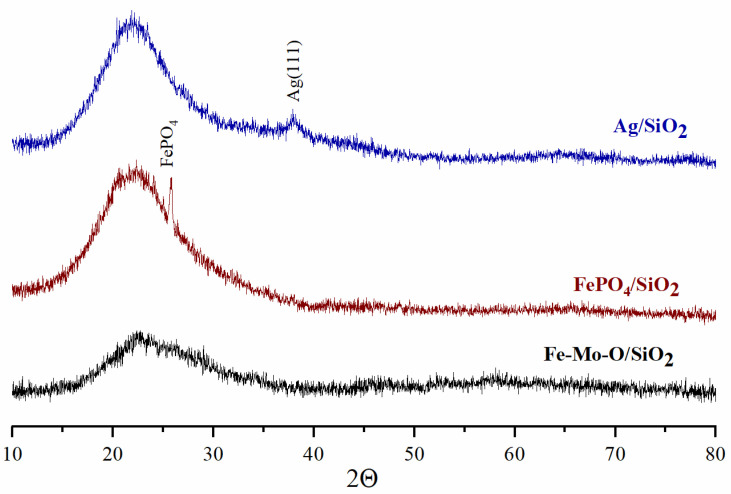
XRD patterns of studied catalysts.

Figure 1 shows the XRD patterns for the supported catalysts and reference samples. The appearance of a wide halo in the range of 2Θ angles up to 30° associated with the amorphous state of SiO_2_ is observed in the XRD patterns for all deposited samples. Figure 1 shows that no crystalline phases are observed by XRD in the Fe-Mo-O/SiO_2_ sample. The XRD pattern of the FePO_4_/SiO_2_ sample features a reflection that corresponds to the FePO_4_ iron orthophosphate phase (No. 01-077-0094). The low-intensity reflection in the range of 38° 2Θ in the XRD pattern for the Ag/SiO_2_ reference sample corresponds to the metallic Ag(111) phase.

**Figure 2:**
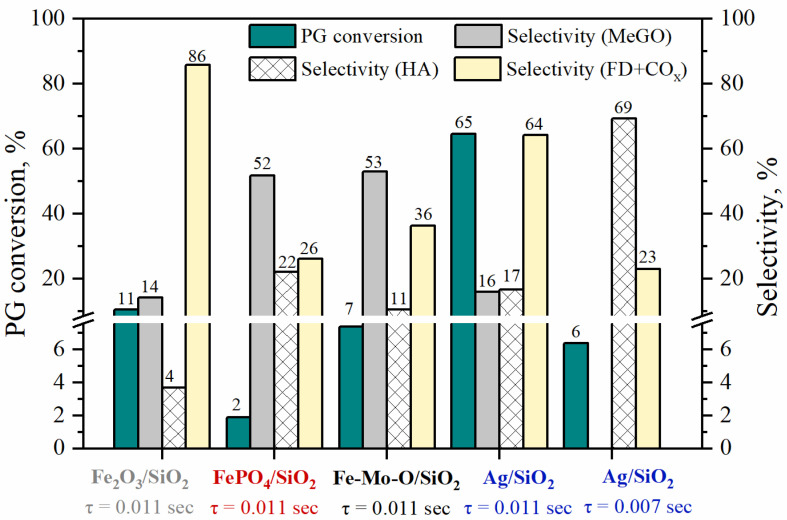
Catalytic properties of prepared catalysts in selective PG oxidation.

The catalytic properties of the prepared catalysts FePO_4_/SiO_2_, Fe-Mo-O/SiO_2_ and Ag/SiO_2_ (reference sample) were studied in the reaction of vapor-phase PG oxidation to methylglyoxal under the same conditions for all catalysts: contact time was 0.011 s (m_cat_ = 0.15 g) and temperature was 350 °C. For the Ag/SiO_2_ sample, the additional catalytic experiments were carried out at a decreased contact time of 0.007 s (m_cat_ = 0.1 g). Figure 2 represents the results obtained.

Thus, in the case of iron-containing catalysts, at the PG conversion of 2 to 7%, the total selectivity towards C_3_ products is ~70%, including ~50% towards methylglyoxal for both FePO_4_/SiO_2_ and Fe-Mo-O/SiO_2_ catalysts. The contribution of the side processes with the cleavage of the C–C bond yielding C_1_ products (formaldehyde, CO_x_) on these catalysts is 26% and 36%, respectively. A Fe_2_O_3_/SiO_2_ reference sample shows higher activity in PG oxidation when compared with samples FePO_4_/SiO_2_ and Fe-Mo-O/SiO_2_. However, in the case of pristine Fe_2_O_3_/SiO_2_ catalyst, the C–C bond cleavage route is the dominant one, and the total selectivity of formation of the C–C bond cleavage products reaches 86% (Figure 2), while the selectivity towards the desired methylglyoxal does not exceed 20%. The use of FePO_4_/SiO_2_ and Fe-Mo-O/SiO_2_ catalysts permits to achieve high selectivity towards methylglyoxal while keeping the PG conversion in the case of Mo introduction in the composition of Fe-containing catalyst.

For the Ag-containing catalyst at a contact time of 0.011 s, an increase in the PG conversion to up to 65% is accompanied by a decrease in the total selectivity towards C_3_ products up to 33% (selectivities towards MeGO and HA are 16% and 17%). For correct comparison of the catalytic properties of the Fe-containing catalysts with Ag/SiO_2_, the additional experiments are carried out for the Ag-containing catalyst with a contact time reduced to 0.007 s. At low PG conversion (6%), hydroxyacetone is the main reaction product with a selectivity of ~70%, and methylglyoxal is not formed at this contact time (Figure 2). The formation of products of C–C bond cleavage under these conditions is reduced to up to 23% that is comparable to iron-containing catalysts.

The iron phosphate catalyst exhibits the lowest activity in PG selective oxidation, while the selectivity towards the target product methylglyoxal reaches 52%, and the selectivity towards by-products does not exceed 30%. For the iron-molybdenum catalyst, a noticeable increase in the PG conversion (up to 7%) is observed, while maintaining high selectivity towards methylglyoxal (Figure 2). Such an effect can be associated with the energies of interaction of the reagents with the surface-active sites of the supported FePO_4_ and Fe_2_(MoO_4_)_3_ catalysts and the structure of the formed intermediate compounds.

Maintaining high selectivity towards C_3_ products (hydroxyacetone) for the silver-containing supported catalyst is possible only if the contact time is reduced by almost a factor of 2 (from 0.011 to 0.007 s). At low contact time and 350 °C, the PG adsorption on the surface of the Ag/SiO_2_ catalyst occurs predominantly in the monodentate configuration with the participation of OH groups on the silica surface [15].

Thus, at low PG conversion (<10%), the iron-containing catalysts are characterized by high selectivity towards C_3_ products, methylglyoxal (50–53%) and hydroxyacetone (up to 22%), while high selectivity is observed on the Ag-containing catalyst only towards hydroxyacetone (~70%), methylglyoxal is not detected in the product composition. Thus, in the case of iron-containing catalysts, PG is adsorbed on the surface mainly in the bidentate configuration that contributes to the selective conversion of both hydroxyl groups with the formation of methylglyoxal. In the case of the Ag-containing catalyst, at low degree of PG conversion (up to 10%), only monodentate intermediates are adsorbed on the surface as evidenced by the high selectivity towards hydroxyacetone. As the contact time increases, PG is adsorbed on the surface of the Ag/SiO_2_ catalyst both in the mono- and bidentate configurations as evidenced by the methylglyoxal appearance in the products. However, an increase in the contact time also leads to the readsorption of products on the surface of the Ag/SiO_2_ catalyst, which is accompanied by high probability of the C–C bond cleavage; with an increase in the PG conversion, the selectivity towards C_1_ products sharply increases.

To substantiate the obtained experimental results, the quantum-chemical calculations of the main transformations of PG and oxygen into C_3_ products were carried out accounting for the binding energies with the Fe-containing sites. The configuration of the active site for each catalyst was chosen based on the obtained phase analysis results and Raman spectroscopy results in Refs. [13,25]. FePO_4_ and Fe_2_(MoO_4_)_3_ structures were used as models of active sites in iron phosphate and iron molybdenum catalysts, respectively.

Results of theoretical calculations show that the first stage of reagent conversion comprises the adsorption and dissociation of molecular oxygen on the active sites of the catalysts (see Supplementary Materials for the profile of interactions of the key reagents, intermediates (Figure S4), and products with the active sites along the reaction coordinate (Tables S1 and S2)). In both cases, the formation of atomic oxygen species adsorbed on Fe sites is observed. The PG is then adsorbed to form adsorbed PDO intermediate through the O–H bond breaking. Moreover, at the iron-phosphate site, a stronger interaction (−715 kJ/mol) occurs between the diol molecule and the Lewis Fe^3+^ center contrary to the iron-molybdenum system (Table 2). In this case, one should expect a decrease in the activity of the iron-phosphate catalyst due to an increase in the residence time of a strongly bound adsorption complex. In the case of the iron–molybdenum catalysts, the PG binding activates the O–H bond both on the Fe-containing sites and on the Fe-O-Mo moiety. The increase of the C–O bond length to up to 1.46 Å in the alcohol molecule (compared to 1.42 Å for the isolated molecule) also occurs at the Fe-containing sites.

The subsequent PDO conversion both at the FePO_4_ and Fe_2_(MoO_4_)_3_ sites occurs via the formation of intermediates, namely, propan-1-al (PPA) and oxopropoxy (OPO). In turn, the PPA and OPO are the intermediates species in the formation of methylglyoxal, hydroxyacetone (and formaldehyde), respectively. The binding energy of the PPA structure (Table 2) is higher for the Fe_2_(MoO_4_)_3_ site due to the assistance of adsorbed atomic oxygen species. The binding energies with the Fe_2_(MoO_4_)_3_ site for the OPO and PPA intermediates are practically similar. However, the presence of the adsorbed oxygen atom strengthens the PPA binding energy with the catalyst surface, while for the OPO structure it results in its decrease. It is noteworthy that in the case of the Fe_2_(MoO_4_)_3_ site, the strengthening of the binding energy with the catalyst surface occurs due to the breaking of the C_2_–H bond followed by the methylglyoxal desorption and the formation of the Fe–O_ads_H bond (O_ads_—oxygen atom adsorbed on the Fe site).

As in the case of PDO intermediate formation, the strength of the OPO binding to the active site is higher for the FePO_4_ site as compared to the Fe_2_(MoO_4_)_3_ site (Table 2). It is noteworthy that the OPO interaction with both FePO_4_ and Fe_2_(MoO_4_)_3_ sites is more favorable in the absence of adsorbed atomic oxygen species. Further transformation of the OPO at the Fe_2_(MoO_4_)_3_ site in the presence of atomic oxygen is accompanied by the elongation of the C_1_–C_2_ bond to up to 1.67 Å (1.59 Å in the isolated species) followed by its cleavage and desorption of the C_1_ products due to the weak interaction with the active site. In this case, the parallel transformations of the OPO intermediate into hydroxyacetone and C_1_ by-products occur depending on the presence of the adsorbed oxygen atom. Moreover, at the FePO_4_ sites, both transformation routes of the OPO intermediate are implemented to similar extent that is also evidenced by the experimental data (Figure 2).

The methylglyoxal formation is more favorable during the PPA dehydrogenation, while hydroxyacetone is formed as a result of the OPO transformations through the oxidative route. Since the reduction of the surface sites in the H_2_-TPR mode for the Fe_2_(MoO_4_)_3_ catalyst [25] proceeds at higher temperatures (by 100 °C higher as compared to FePO_4_ [24]) indicating higher strength of oxygen binding to the surface, the dehydrogenation route is more favorable. In the presence of the adsorbed oxygen atom, a competing process for the OPO selective oxidation to HA is the C–C bond cleavage in the OPO species yielding formaldehyde. At the same time, it is noteworthy that the oxygen presence in the reaction medium favorably affects the course of the oxidative processes as was shown in Ref. [29] exemplified by the oxidation of organic compounds (acrolein, formaldehyde, ethanol, etc.) on the oxide V–Ti catalysts.

Thus, the studied systems are characterized by a relatively similar position of the main intermediates formed during the PG oxidation. However, significant differences are found in the interaction energies of the key PDO intermediate with the active sites of the FePO_4_ and Fe_2_(MoO_4_)_3_. The PDO binding energy on the iron-phosphate site is much lower and amounts to −586 kJ/mol (−715.5 kJ/mol in the presence of atomic oxygen). In this case, the strong PDO interaction with the FePO_4_ site leads to the increase of the residence time of the adsorbed intermediates on the active sites of the catalyst and, accordingly, to a decrease in the degree of PG conversion, which is consistent with the catalytic experiments. According to the results of theoretical calculations, on the iron-molybdenum site, an intermediate value of the PDO binding energy was obtained as compared to those over FePO_4_. Thus, it can be assumed that the use of iron-molybdenum catalysts leads to an increase in the turnover frequency for the Fe-containing sites upon interaction with the intermediate compounds and, at the same time, facilitates further selective oxidation of PG without breaking the C–C bond.

## 4. Conclusions

In the present work, the experimental and theoretical studies of the catalytic properties of FePO_4_/SiO_2_ and Fe-Mo-O/SiO_2_ catalysts in the selective oxidation of propylene glycol to methylglyoxal were carried out. The experimental comparison of the catalytic properties of the supported iron-containing catalysts with the silver-containing one was also carried out using the Ag/SiO_2_ reference sample. It was shown that the iron-containing catalysts FePO_4_/SiO_2_ and Fe-Mo-O/SiO_2_ were highly selective in the propylene glycol oxidation to methylglyoxal. While the Ag-containing sample was highly active, however, the composition of the reaction products was dominated by the contribution of the by-products of the C–C bond cleavage. Moreover, with a comparable propylene glycol conversion over all catalysts (up to 10%), for FePO_4_ and Fe-Mo-O species the formation of the bidentate-bound intermediate with the methylglyoxal release was favorable. The results of the catalytic experiments were consistent with the theoretical calculations carried out for the main reactions of the propylene glycol oxidation to methylglyoxal at the FePO_4_ and Fe_2_(MoO_4_)_3_ sites. In accordance with the results obtained, it can be concluded that the supported iron-containing catalysts can be an alternative to conventional oxidation catalysts based on noble metals.

## Figures and Tables

**Table 1:** Physical-chemical characteristics of prepared catalysts.

Sample	Fe_2_O_3_, wt.%	[P_2_O_5_ or MoO_3_], wt.%	[P or Mo]/Fe, mol.	S_BET_, m^2^/g
FePO_4_/SiO_2_	2.4	5.1	2.4	289
Fe-Mo-O/SiO_2_	2.5	8.7	1.9	240
SiO_2_ support	-	-	-	300

**Table 2:** Calculated binding energies (kJ/mol) of key intermediates with model active sites of studied catalysts.

Intermediates	FePO_4_ + O_ads_	FePO_4_	Fe_2_(MoO_4_)_3_ + O_ads_	Fe_2_(MoO_4_)_3_
PDO	−715	−586	−576	−549
OPO	−360	−498	−284	−422
PPA	−439	−389	−480	−408

## Data Availability

The data presented in this study are available on request from the corresponding author.

## Supplementary Materials

The following supporting information is available online at https://www.mdpi.com/article/10.3390/ma15051906/s1, Figure S1: Geometries of substrates; Figure S2: Calculated structure of iron phosphate (FePO4H); Table S1: DFT-optimized cartesian coordinates for the main FePO_4_-based models; Figure S3: Calculated structure of iron molybdate (Fe_2_(MoO_4_)_3_); Table S2: DFT-optimized cartesian coordinates for the main Fe_2_(MoO_4_)_3_-based models; Figure S4: Interactions of key reagents, intermediates and products with active sites along the reaction coordinate (B3LYP/DGDZVP level of theory).
