# Supplementary material for: Modifier Effect in Silica-Supported FePO4 and Fe-Mo-O Catalysts for Propylene Glycol Oxidation
Source: Materials (Basel). 2022 Mar 4;15(5):1906. doi: 10.3390/ma15051906 (PMC8911785; doi:10.3390/ma15051906)
Supplement: Supplementary file 1 [file materials-15-01906-s001.zip › materials-1587438-SI.pdf]

*materials*

*Supplementary*

# Modifier Effect in Silica-Supported $FePO_4$ and Fe-Mo-O Catalysts for Propylene Glycol Oxidation

**Darya Y. Savenko [1], Mikhail A. Salaev [1], Valerii V. Dutov [1], Sergei A. Kulinich [2,3] and Olga V. Vodyankina [1,*]**

[1] Laboratory of Catalytic Research, Tomsk State University, 36 Lenin Avenue, 634050 Tomsk, Russia; darya_ebert@mail.ru (D.Y.S.); mihan555@yandex.ru (M.A.S.); dutov_valeriy@mail.ru (V.V.D.)
[2] Research Institute of Science & Technology, Tokai University, Hiratsuka 259-1292, Kanagawa, Japan; skulinich@tokai-u.jp
[3] School of Natural Sciences, Far Eastern Federal University, 690091 Vladivostok, Russia
* Correspondence: vodyankina_o@mail.ru

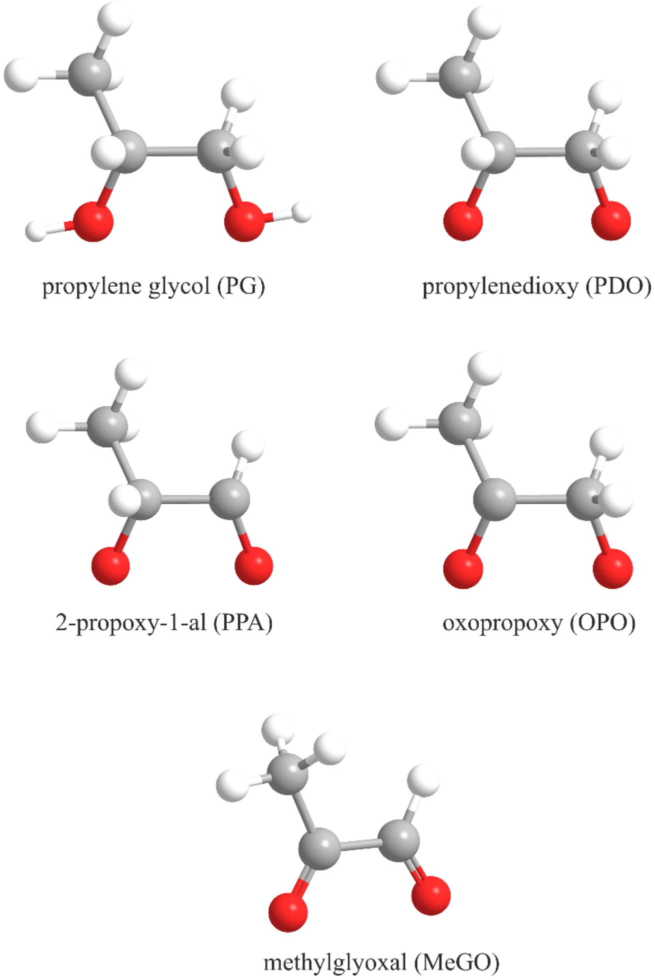


**Figure S1.** Geometries of substrates.

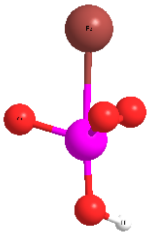

**Figure S2.** Calculated structure of iron phosphate ($FePO_4H$).

**Table S1.** DFT-optimized Cartesian coordinates for the main $FePO_4$–based models.

| Element | X | Y | Z |
|---|---|---|---|
| | | $FePO_4H$ | |
| Fe | –6.095141 | –0.493745 | –1.068161 |
| P | –8.084016 | 0.186545 | –1.289914 |
| O | –9.583237 | 1.287554 | –1.021465 |
| O | –7.445038 | 0.695499 | –1.450785 |
| O | –7.445038 | –0.584438 | –2.504909 |
| O | –7.793282 | –0.825541 | –0.119970 |
| H | –9.660208 | 1.665827 | –1.414896 |
| | | $FePO_4H+O_2$ | |
| Fe | –0.482871 | –1.860748 | –0.138670 |
| P | –2.501492 | –1.065480 | 0.116941 |
| O | –1.363558 | –1.065480 | 0.305461 |
| O | –4.002781 | –0.574747 | 0.131324 |
| O | –1.936945 | –1.732065 | –1.251926 |
| O | –2.139232 | –2.231328 | 1.067269 |
| H | –4.137853 | 0.348205 | –0.153127 |
| O | 0.803925 | –2.807090 | 0.633210 |
| O | 1.235807 | –1.598708 | 0.220019 |
| | | $FePO_4H+O$ | |
| Fe | 1.488834 | 0.146322 | 0.331962 |
| P | –0.567447 | 0.880776 | 0.102574 |
| O | 0.563688 | 1.939321 | 0.376228 |
| O | –2.052713 | 1.383365 | –0.063492 |
| O | 0.100124 | 0.100582 | –1.089412 |
| O | –0.251167 | –0.137468 | 1.257528 |
| H | –2.159088 | 2.352304 | –0.025212 |
| O | 2.917369 | –0.387401 | 0.487925 |
| | | $FePO_4H+PG$ | |
| C | 1.571481 | –0.495553 | –0.029929 |
| C | 1.441737 | 0.962265 | 0.394182 |
| C | 2.098614 | 1.924530 | –0.585656 |
| O | 0.800839 | –1.270212 | 0.927829 |
| O | 0.003111 | 1.189987 | 0.463575 |
| H | 2.619547 | –0.809562 | 0.004743 |
| H | 1.151500 | –0.641255 | –1.029846 |
| H | 1.860847 | 1.080216 | 1.402027 |
| H | 1.961805 | 2.961124 | –0.260028 |
| H | 3.176075 | 1.737718 | –0.641895 |
| H | 1.666148 | 1.815342 | –1.584078 |
| H | 0.650792 | –2.172383 | 0.585255 |
| H | –0.213111 | 2.079279 | 0.787550 |
| Fe | –1.068256 | –0.497643 | 0.879737 |
| P | –2.723857 | –1.451155 | –0.118861 |
| O | –3.114542 | –0.079440 | 0.501274 |
| O | –3.913531 | –2.197056 | –0.905439 |
| O | –1.451781 | –1.123464 | –0.993036 |
| O | –2.086522 | –2.259924 | 1.056698 |
| H | –4.699197 | –1.626212 | –0.961303 |
| | | $FePO_4H+O+PG$ | |
| C | 2.324294 | 0.835031 | 0.133313 |
| C | 2.401245 | 2.308005 | 0.548118 |
| C | 1.210433 | 3.157593 | 0.115396 |
| O | 1.412605 | 0.139719 | 1.044258 |
| O | 2.534716 | 2.248188 | 1.988590 |
| H | 3.296713 | 0.346316 | 0.234695 |

| | | | |
|---|---|---|---|
| H | 1.929450 | 0.706096 | –0.873623 |
| H | 3.326143 | 2.716190 | 0.118977 |
| H | 1.344825 | 4.192215 | 0.453369 |
| H | 1.126606 | 3.177358 | –0.974931 |
| H | 0.261669 | 2.784894 | 0.509003 |
| H | 1.475623 | 0.603476 | 1.911025 |
| H | 2.280455 | 3.105016 | 2.366528 |
| Fe | –0.145572 | –0.821914 | 0.544418 |
| P | –1.859385 | 0.646332 | –0.581660 |
| O | –2.048805 | 2.101522 | –0.791765 |
| O | –3.057556 | –0.236502 | –1.215859 |
| O | –0.479385 | –0.020592 | –1.082337 |
| O | –1.658058 | 0.123250 | 0.937200 |
| H | –3.728278 | 0.343862 | –1.614928 |
| O | –0.120837 | –2.356057 | 0.753114 |
| | | $FePO_4H$+PDO | |
| C | –3.681492 | –2.700196 | –0.901429 |
| C | –2.850783 | –1.416872 | –0.902028 |
| C | –2.114959 | –1.152360 | –2.219511 |
| O | –4.993384 | –2.343711 | –1.373071 |
| O | –3.804839 | –0.356335 | –0.659984 |
| H | –3.790314 | –3.100634 | 0.114714 |
| H | –3.293671 | –3.494102 | –1.549744 |
| H | –2.136616 | –1.378783 | –0.069804 |
| H | –1.670267 | –0.154039 | –2.206564 |
| H | –1.315347 | –1.888218 | –2.353283 |
| H | –2.801967 | –1.219488 | –3.068841 |
| Fe | –5.438581 | –0.689082 | –1.123316 |
| P | –7.558075 | 0.703565 | –1.322407 |
| O | –7.871917 | 2.126155 | –1.096107 |
| O | –8.823595 | –0.174504 | –1.801731 |
| O | –6.348381 | 0.308550 | –2.329083 |
| O | –6.871095 | –0.176410 | –0.144345 |
| H | –9.617118 | 0.382964 | –1.884165 |
| | | $FePO_4H$+O+PDO | |
| C | –0.459017 | 0.888779 | –2.332791 |
| C | –0.186257 | –0.590793 | –2.574791 |
| C | 1.166262 | –0.777661 | –3.286328 |
| O | –0.834161 | 1.395460 | –1.288915 |
| O | –0.193735 | –1.328856 | –1.378071 |
| H | –1.770602 | 1.049327 | 0.182562 |
| H | –0.298563 | 1.533408 | –3.221856 |
| H | –0.986433 | –0.908662 | –3.266889 |
| H | 1.297519 | –1.834711 | –3.528071 |
| H | 1.212523 | –0.198469 | –4.214650 |
| H | 1.984729 | –0.468783 | –2.629873 |
| Fe | –1.586357 | –2.300316 | –0.917369 |
| P | –2.864439 | –0.771947 | 0.582904 |
| O | –2.449154 | 0.737970 | 0.840092 |
| O | –4.178908 | –0.915679 | 1.466591 |
| O | –3.049004 | –1.064356 | –0.939679 |
| O | –1.805981 | –1.836768 | 0.954188 |
| H | –4.570118 | –0.065648 | 1.735070 |
| O | –1.672805 | –3.747596 | –1.481024 |
| | | $FePO_4H$+OPO | |
| C | 1.542085 | –1.130946 | –0.870611 |
| C | 1.488932 | 0.313012 | –1.298646 |

| | | | |
|---|---|---|---|
| C | 2.492617 | 0.915020 | –2.230360 |
| O | 0.552659 | –1.385564 | 0.078405 |
| O | 0.568168 | 0.998099 | –0.814370 |
| H | 2.546435 | –1.363884 | –0.477807 |
| H | 1.410416 | –1.764910 | –1.765246 |
| H | 3.342630 | 1.270269 | –1.633697 |
| H | 2.873304 | 0.177721 | –2.942012 |
| H | 2.061902 | 1.772015 | –2.751285 |
| Fe | –0.583501 | –0.046117 | 0.472423 |
| P | –2.675754 | 0.334950 | 1.834458 |
| O | –2.957288 | 0.870837 | 3.182499 |
| O | –4.022535 | –0.054057 | 1.020191 |
| O | –1.807389 | 1.242311 | 0.802493 |
| O | –1.687243 | –0.918007 | 1.647566 |
| H | –4.806438 | 0.183751 | 1.544597 |
| $FePO_4H$+O+OPO | | | |
| C | 0.429744 | –0.875519 | –1.439907 |
| C | 0.538447 | 0.631384 | –1.500067 |
| C | 0.797175 | 1.326431 | –2.798945 |
| O | 0.152702 | –1.322695 | –0.136550 |
| O | 0.364844 | 1.270170 | –0.451957 |
| H | 1.363923 | –1.329700 | –1.805143 |
| H | –0.380036 | –1.173523 | –2.122704 |
| H | 1.224585 | 2.315283 | –2.624559 |
| H | 1.439144 | 0.734735 | –3.456827 |
| H | –0.175411 | 1.448567 | –3.292323 |
| Fe | –0.210733 | –0.047964 | 1.049385 |
| P | –3.049414 | 0.109326 | –0.177905 |
| O | –2.066401 | 0.114525 | 1.046466 |
| O | –4.264217 | 1.042623 | 0.316737 |
| O | –2.426106 | 0.581480 | –1.492121 |
| O | –3.589347 | –1.281161 | –0.569067 |
| H | –4.069315 | 1.489157 | 1.158112 |
| O | 0.780017 | 0.504382 | 2.124575 |
| $FePO_4H$+PPA | | | |
| C | 1.403431 | –1.568002 | 0.097462 |
| C | 1.909997 | –0.198159 | 0.415298 |
| C | 2.877300 | 0.313549 | –0.672220 |
| O | 0.180347 | –1.745703 | –0.006756 |
| O | 0.814958 | 0.669622 | 0.583675 |
| H | 2.470435 | –0.277329 | 1.365423 |
| H | 2.076472 | –2.425757 | –0.050585 |
| H | 3.204045 | 1.316504 | –0.389559 |
| H | 3.757210 | –0.331980 | –0.764386 |
| H | 2.368443 | 0.375007 | –1.638041 |
| Fe | –0.799962 | –0.077288 | 0.331791 |
| P | –2.887458 | 0.515553 | 0.083466 |
| O | –2.015467 | 1.644969 | 0.670440 |
| O | –4.423540 | 0.816747 | –0.203274 |
| O | –2.010712 | 0.061182 | –1.171443 |
| O | –2.597719 | –0.713136 | 1.007749 |
| H | –4.623280 | 1.768522 | –0.256242 |
| $FePO_4H$+O+PPA | | | |
| C | 1.609983 | –1.503286 | 0.047330 |
| C | 1.807717 | –0.027721 | 0.221455 |
| C | 2.327718 | 0.645560 | –1.060145 |
| O | 0.520372 | –2.004647 | 0.321531 |

| | | | |
|---|---|---|---|
| O | 0.588270 | 0.561062 | 0.633218 |
| H | 2.557785 | 0.098958 | 1.023002 |
| H | 2.426597 | –2.144590 | –0.319546 |
| H | 2.430660 | 1.716696 | –0.874113 |
| H | 3.306992 | 0.245028 | –1.341547 |
| H | 1.627520 | 0.498995 | –1.887044 |
| Fe | –0.734357 | –0.531966 | 1.064178 |
| P | –3.011003 | 0.284988 | 0.259670 |
| O | –1.937511 | 0.884703 | 1.284660 |
| O | –2.967912 | 1.245770 | –1.039503 |
| O | –2.005902 | –0.992051 | –0.081719 |
| O | –4.386832 | –0.058399 | 0.642759 |
| H | –2.130534 | 1.735796 | –1.110937 |
| O | –0.755263 | –1.181596 | 2.489952 |
| $FePO_4H$+MeGO | | | |
| C | 2.337906 | –1.972785 | 1.203490 |
| C | 2.664092 | –0.780015 | 2.008821 |
| C | 3.932418 | –0.622316 | 2.785457 |
| O | 1.215642 | –1.958747 | 0.647581 |
| O | 1.751225 | 0.083621 | 2.016184 |
| H | 2.986921 | –2.850548 | 1.119227 |
| H | 3.874743 | 0.266563 | 3.415006 |
| H | 4.115610 | –1.504814 | 3.408462 |
| H | 4.785366 | –0.525374 | 2.102416 |
| Fe | 0.229903 | –0.317709 | 0.864067 |
| P | –1.880250 | 0.062573 | 1.036549 |
| O | –0.930815 | 1.310499 | 1.088656 |
| O | –3.447937 | 0.330917 | 1.174734 |
| O | –1.432512 | –0.657125 | –0.266828 |
| O | –1.209096 | –0.846075 | 2.140634 |
| H | –3.658917 | 1.024335 | 1.824543 |
| $FePO_4H$+O+MeGO | | | |
| Fe | 1.004139 | –1.262654 | 0.121720 |
| P | –1.447555 | –0.768061 | –0.161707 |
| O | –2.334925 | 0.412828 | –0.186552 |
| O | –2.245311 | –2.160842 | –0.374626 |
| O | –0.186115 | –0.822137 | –1.181348 |
| O | –0.489247 | –1.001547 | 1.126983 |
| H | –3.196208 | –1.989040 | –0.486608 |
| O | 1.688913 | –2.659747 | 0.103141 |
| C | 3.363854 | 0.342843 | 0.556816 |
| O | 2.144094 | 0.205708 | 0.385499 |
| C | 3.876447 | 1.786460 | 0.736094 |
| O | 3.138527 | 2.739675 | 0.712801 |
| C | 4.359987 | –0.765292 | 0.602090 |
| H | 4.970932 | 1.874083 | 0.886911 |
| H | 3.893072 | –1.741139 | 0.466409 |
| H | 5.118119 | –0.602245 | –0.175248 |
| H | 4.891578 | –0.735192 | 1.562324 |
| $FePO_4H$+HA | | | |
| C | 1.528555 | –1.175981 | –0.852258 |
| C | 1.514359 | 0.324060 | –0.527125 |
| C | 1.317957 | 1.305725 | –1.642048 |
| O | 0.941786 | –1.806070 | 0.323467 |
| O | 1.805338 | 0.678550 | 0.651419 |
| H | 2.549918 | –1.550162 | –0.986546 |
| H | 0.925221 | –1.383329 | –1.737200 |

| | | | |
|---|---|---|---|
| H | 1.468848 | 2.322398 | –1.276999 |
| H | 2.021951 | 1.093326 | –2.457281 |
| H | 0.301546 | 1.199411 | –2.035566 |
| Fe | 0.020505 | –0.144050 | 1.004134 |
| P | –2.102991 | 0.262157 | 0.862599 |
| O | –1.320872 | 1.449246 | 1.472172 |
| O | –3.680042 | 0.465766 | 0.681508 |
| O | –1.316545 | –0.044269 | –0.478146 |
| O | –1.653807 | –0.980934 | 1.704457 |
| H | –3.974475 | 1.330337 | 1.018360 |
| H | 0.229749 | –2.417282 | 0.060053 |
| | | $FePO_4H$+O+HA | |
| C | 0.037142 | –0.523879 | –2.236572 |
| C | 0.763427 | 0.603409 | –1.524527 |
| C | 1.790215 | 1.379926 | –2.291718 |
| O | –0.323107 | –1.549415 | –1.361910 |
| O | 0.566987 | 0.897862 | –0.332406 |
| H | 0.705112 | –0.894426 | –3.028603 |
| H | –0.823693 | –0.051935 | –2.737729 |
| H | 2.013404 | 2.327251 | –1.800121 |
| H | 2.706611 | 0.778662 | –2.348704 |
| H | 1.452880 | 1.545830 | –3.320115 |
| Fe | –0.831383 | 0.596270 | 0.909875 |
| P | –2.945497 | –0.494955 | 0.080650 |
| O | –2.200970 | 0.882542 | –0.296309 |
| O | –4.370904 | –0.189985 | 0.753431 |
| O | –3.083898 | –1.474446 | –1.030273 |
| O | –1.879642 | –0.861484 | 1.239032 |
| H | –4.319114 | 0.421740 | 1.509022 |
| O | –0.605449 | 1.474720 | 2.172243 |
| H | –1.294521 | –1.721887 | –1.410065 |
| | | $FePO_4H$+FD | |
| Fe | 0.218522 | –1.058340 | 0.549046 |
| P | –1.665241 | –0.278368 | –0.105437 |
| O | –0.888688 | 0.523798 | 1.017000 |
| O | –3.045778 | 0.350110 | –0.609484 |
| O | –0.507158 | –0.398324 | –1.178436 |
| O | –1.769405 | –1.704915 | 0.475524 |
| H | –2.955040 | 1.248384 | –0.972019 |
| C | 2.117778 | 0.906638 | 0.053598 |
| O | 1.951329 | –0.231800 | 0.502787 |
| H | 3.130441 | 1.330079 | 0.052160 |
| H | 1.268440 | 1.480438 | –0.335139 |

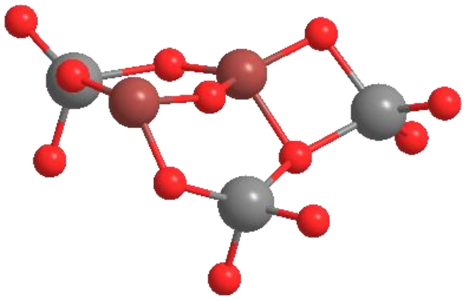

**Figure S3.** Calculated structure of iron molybdate ($Fe_2(MoO_4)_3$).

**Table S2.** DFT-optimized Cartesian coordinates for the main $Fe_2(MoO_4)_3$–based models.

| Element | X | Y | Z |
|---|---|---|---|
| | | $Fe_2(MoO_4)_3$ | |
| Mo | 0.174485 | –1.731996 | 0.936697 |
| Fe | 3.398447 | –1.877739 | 0.543483 |
| O | –0.466200 | –1.326343 | –0.602810 |
| O | 1.982887 | –1.023392 | 1.068315 |
| O | –0.848912 | –1.082973 | 2.145065 |
| O | 0.307871 | –3.625066 | 1.084238 |
| Mo | 5.826907 | –1.380550 | –0.624543 |
| O | 6.383842 | 0.183185 | –1.034499 |
| O | 7.094858 | –2.494315 | –0.899858 |
| O | 5.064077 | –1.508090 | 1.123159 |
| O | 4.029335 | –1.862803 | –1.292628 |
| Mo | 3.030129 | –3.548249 | –2.123999 |
| O | 2.190309 | –2.734460 | –3.367425 |
| O | 2.830207 | –3.440750 | 0.085816 |
| O | 1.781780 | –4.890835 | –1.487876 |
| O | 4.320574 | –4.394877 | –2.857430 |
| Fe | 1.507505 | –4.610568 | 0.241806 |
| | | $Fe_2(MoO_4)_3+O_2$ | |
| Mo | –2.488270 | 0.675963 | 0.963609 |
| Fe | 0.149837 | 0.162428 | 2.265839 |
| O | –2.529429 | 1.850446 | –0.268175 |
| O | –1.245758 | 1.113630 | 2.485388 |
| O | –3.986794 | 0.795167 | 1.781313 |
| O | –2.622879 | –1.219138 | 0.301480 |
| Mo | 2.260370 | 1.241070 | 0.079654 |
| O | 1.317726 | 2.506462 | –0.573796 |
| O | 3.863122 | 1.818909 | 0.187315 |
| O | 1.678189 | 0.866436 | 1.896537 |
| O | 2.225493 | –0.292126 | –1.071862 |
| Mo | 0.946004 | –1.236269 | –2.136796 |
| O | –0.006922 | –0.143137 | –3.039873 |
| O | –0.381399 | –0.188185 | 0.558195 |
| O | –0.208133 | –2.362786 | –1.056675 |
| O | 1.751634 | –2.245699 | –3.253955 |
| Fe | –1.050928 | –1.871904 | 0.363499 |
| O | –0.542208 | –2.410792 | 1.918030 |
| O | 0.018945 | –1.470496 | 2.796083 |
| | | $Fe_2(MoO_4)_3+O$ | |
| Mo | –2.678957 | 0.451253 | 1.025184 |
| Fe | 0.504555 | 0.273838 | 1.266690 |
| O | –2.692653 | 0.908153 | –0.633724 |
| O | –0.922074 | 0.992646 | 1.799728 |
| O | –4.043579 | 1.086332 | 1.839257 |
| O | –2.501575 | –1.392615 | 1.210151 |
| Mo | 2.587862 | 1.597827 | 0.123803 |
| O | 2.559277 | 3.300785 | 0.029417 |
| O | 4.119602 | 1.071145 | –0.406475 |
| O | 2.050658 | 0.904676 | 1.846872 |
| O | 0.974140 | 0.670310 | –0.544246 |
| Mo | 0.433467 | –1.107208 | –1.593380 |
| O | 0.034393 | –0.355297 | –3.075460 |
| O | 0.283929 | –1.348552 | 0.697239 |
| O | –1.030138 | –2.260647 | –1.215636 |
| O | 1.855748 | –2.007586 | –1.888040 |

| | | | |
|---|---|---|---|
| Fe | –1.206457 | –2.471774 | 0.566687 |
| O | –1.174199 | –3.912289 | 1.118934 |
| | | $Fe_2(MoO_4)_3$+PG | |
| Mo | –3.662732 | 2.009633 | –9.536199 |
| Fe | –0.616234 | 1.511831 | –9.876891 |
| O | –3.647085 | 2.463537 | –11.231549 |
| O | –1.909396 | 2.261666 | –8.860418 |
| O | –4.834389 | 2.886279 | –8.644615 |
| O | –3.947484 | 0.159412 | –9.455362 |
| Mo | 0.939267 | 3.070825 | –11.507449 |
| O | 1.190786 | 4.740390 | –11.201487 |
| O | 1.965365 | 2.598412 | –12.794958 |
| O | 1.133402 | 1.996013 | –9.923537 |
| O | –0.917764 | 2.556477 | –11.612153 |
| Mo | –2.174680 | 1.242798 | –12.787613 |
| O | –2.986793 | 2.153251 | –13.982938 |
| O | –1.436962 | 0.299920 | –10.749843 |
| O | –3.263446 | –0.244905 | –12.273474 |
| O | –0.864255 | 0.526642 | –13.639409 |
| Fe | –2.813997 | –0.785429 | –10.584845 |
| C | –0.853096 | –0.436949 | –7.453808 |
| C | –1.261101 | –1.824378 | –7.973519 |
| C | –1.519402 | –2.803071 | –6.830127 |
| O | –0.034899 | 0.259499 | –8.427467 |
| O | –2.454137 | –1.774090 | –8.819076 |
| H | –0.259222 | –0.554868 | –6.544041 |
| H | –1.724889 | 0.182591 | –7.225067 |
| H | –0.475986 | –2.198214 | –8.633551 |
| H | –1.872702 | –3.756907 | –7.228500 |
| H | –0.599213 | –2.983832 | –6.266287 |
| H | –2.273536 | –2.418406 | –6.133097 |
| H | 0.765906 | 0.645529 | –8.030176 |
| H | –3.199126 | –1.411256 | –8.295643 |
| | | $Fe_2(MoO_4)_3$+O+PG | |
| Mo | –2.684097 | 1.793588 | –1.037340 |
| Fe | –0.154765 | 0.920673 | 0.656558 |
| O | –3.553059 | 3.130982 | –1.655762 |
| O | –1.187288 | 2.282451 | –0.080869 |
| O | –3.668620 | 0.871549 | 0.098288 |
| O | –2.057526 | 0.580279 | –2.400278 |
| Mo | 2.250627 | 2.286917 | –1.041124 |
| O | 1.221190 | 3.111780 | –2.142163 |
| O | 3.632235 | 3.257538 | –0.750923 |
| O | 1.373469 | 1.908193 | 0.569921 |
| O | 2.702060 | 0.549091 | –1.807794 |
| Mo | 2.046782 | –1.219698 | –1.902517 |
| O | 3.122305 | –2.108532 | –2.882769 |
| O | 0.045709 | –0.062407 | –0.713525 |
| O | 0.333838 | –1.355546 | –2.781446 |
| O | 2.060739 | –1.956933 | –0.318396 |
| Fe | –1.074139 | –0.773385 | –1.871365 |
| O | –1.872680 | –1.917982 | –1.151574 |
| C | –1.412515 | –1.221292 | 2.052143 |
| C | –0.010095 | –1.229980 | 2.655054 |
| C | 0.533804 | –2.625857 | 2.916888 |
| O | –1.645172 | 0.153018 | 1.628829 |
| O | 0.869325 | –0.472986 | 1.763765 |

| | | | |
|---|---|---|---|
| H | –2.166350 | –1.482944 | 2.800013 |
| H | –1.497968 | –1.876044 | 1.177607 |
| H | –0.020757 | –0.640675 | 3.576432 |
| H | 1.532828 | –2.563341 | 3.355771 |
| H | –0.114844 | –3.154285 | 3.622730 |
| H | 0.592436 | –3.212842 | 1.995049 |
| H | –2.556769 | 0.306407 | 1.214537 |
| H | 1.332295 | –1.077234 | 1.124657 |
| | $Fe_2(MoO_4)_3$+PDO | | |
| Mo | –3.012801 | 2.739331 | –0.612618 |
| Fe | –0.312182 | 2.240176 | 0.777085 |
| O | –2.588751 | 2.788664 | –2.273617 |
| O | –1.581826 | 3.397927 | 0.516825 |
| O | –4.380558 | 3.738644 | –0.392402 |
| O | –3.454214 | 0.935671 | –0.074274 |
| Mo | 1.908883 | 2.750462 | –1.598216 |
| O | 1.695149 | 4.308695 | –2.273635 |
| O | 3.584879 | 2.399288 | –1.609482 |
| O | 1.269171 | 2.687526 | 0.223984 |
| O | 0.891401 | 1.463168 | –2.551151 |
| Mo | 0.084829 | –0.269283 | –2.225241 |
| O | 0.080073 | –0.966608 | –3.783610 |
| O | –0.800397 | 1.013497 | –0.425345 |
| O | –1.712787 | –0.914836 | –1.609653 |
| O | 1.208144 | –1.171046 | –1.294787 |
| Fe | –2.130205 | –0.194048 | –0.122102 |
| C | –0.253031 | 0.322513 | 2.880387 |
| C | –0.313992 | –0.842745 | 1.885623 |
| C | –0.000859 | –2.168141 | 2.586828 |
| O | –0.498057 | 1.627550 | 2.369284 |
| O | –1.623709 | –0.984706 | 1.306874 |
| H | 0.739164 | 0.322930 | 3.349132 |
| H | –1.010652 | 0.181515 | 3.662479 |
| H | 0.412771 | –0.680198 | 1.082312 |
| H | –0.047935 | –2.985870 | 1.864439 |
| H | 1.007928 | –2.140122 | 3.010586 |
| H | –0.719638 | –2.366852 | 3.386993 |
| | $Fe_2(MoO_4)_3$+O+PDO | | |
| Mo | –2.409227 | 1.807421 | 0.211632 |
| Fe | 0.675831 | 1.711800 | 0.063047 |
| O | –2.894177 | 2.380199 | –1.324856 |
| O | –0.627023 | 2.745369 | 0.328399 |
| O | –3.396708 | 2.627773 | 1.338897 |
| O | –3.127051 | –0.063881 | 0.138109 |
| Mo | 2.538633 | 2.894692 | –1.652813 |
| O | 2.262379 | 4.283517 | –2.619846 |
| O | 4.182171 | 2.434743 | –1.834751 |
| O | 2.030996 | 3.096714 | 0.105251 |
| O | 1.152932 | 1.408466 | –1.677903 |
| Mo | –0.122527 | –0.003421 | –2.564743 |
| O | –0.491187 | 0.869946 | –3.985585 |
| O | –0.699145 | 0.301489 | –0.548953 |
| O | –1.752875 | –1.110008 | –2.248459 |
| O | 1.055436 | –1.182718 | –2.952799 |
| Fe | –1.954329 | –1.080713 | –0.551514 |

| | | | |
|---|---|---|---|
| C | 0.867462 | −0.092756 | 2.227116 |
| C | −0.571100 | 0.033727 | 2.748833 |
| C | −0.894715 | −0.768419 | 3.980986 |
| O | 1.361455 | 0.974863 | 1.473037 |
| O | −1.407387 | 0.742041 | 2.207597 |
| H | 0.941054 | −1.047348 | 1.683330 |
| H | 1.537495 | −0.184060 | 3.093798 |
| H | −0.289000 | −2.180879 | 0.573609 |
| H | −1.971652 | −0.922833 | 4.055721 |
| H | −0.366079 | −1.726794 | 3.989625 |
| H | −0.556871 | −0.201177 | 4.857808 |
| O | −1.229987 | −2.344656 | 0.372224 |
| | $Fe_2(MoO_4)_3$+OPO | | |
| Mo | −2.916784 | 2.426135 | −0.303774 |
| Fe | 0.089997 | 1.933769 | 0.589376 |
| O | −2.885907 | 3.090449 | −1.885046 |
| O | −1.312843 | 2.944779 | 0.691944 |
| O | −4.331242 | 2.994290 | 0.477706 |
| O | −2.887897 | 0.539657 | −0.352564 |
| Mo | 1.875323 | 2.114273 | −1.969587 |
| O | 0.747672 | 2.901093 | −2.984483 |
| O | 3.383999 | 2.853897 | −2.278332 |
| O | 1.556822 | 2.569373 | −0.078415 |
| O | 2.085263 | 0.276256 | −2.485063 |
| Mo | 1.322031 | −1.464967 | −2.238151 |
| O | 1.799228 | −2.543139 | −3.480272 |
| O | −0.172949 | 0.945110 | −0.856840 |
| O | −0.542035 | −1.355409 | −2.153024 |
| O | 1.866735 | −2.080630 | −0.707523 |
| Fe | −1.388176 | −0.332633 | −0.891894 |
| C | 0.724600 | −0.538045 | 1.849995 |
| C | −0.350001 | −1.583049 | 1.553920 |
| C | −0.343635 | −2.831196 | 2.376355 |
| O | 0.279161 | 0.795099 | 1.901049 |
| O | −1.197000 | −1.460664 | 0.654406 |
| H | 1.506986 | −0.705509 | 1.095046 |
| H | 1.162979 | −0.758140 | 2.830946 |
| H | −1.049133 | −3.563476 | 1.984172 |
| H | 0.671158 | −3.246729 | 2.386613 |
| H | −0.596350 | −2.578593 | 3.413439 |
| | $Fe_2(MoO_4)_3$+O+OPO | | |
| Mo | −2.904336 | 2.358035 | −0.242391 |
| Fe | 0.403554 | 1.749076 | 0.032619 |
| O | −3.824589 | 3.283448 | −1.351455 |
| O | −1.076427 | 2.709726 | −0.413351 |
| O | −3.484937 | 2.697538 | 1.336901 |
| O | −3.080173 | 0.445942 | −0.636051 |
| Mo | 2.421537 | 2.973625 | −1.435910 |
| O | 2.158070 | 4.272327 | −2.521920 |
| O | 4.115157 | 2.767756 | −1.262695 |
| O | 1.553177 | 3.202718 | 0.223603 |
| O | 1.388611 | 1.335550 | −1.748099 |
| Mo | 0.390811 | −0.287562 | −2.481139 |
| O | 0.041880 | 0.291465 | −4.052254 |
| O | −0.326327 | 0.065527 | −0.485749 |

| | | | |
|---|---|---|---|
| O | –1.315074 | –1.311329 | –2.153477 |
| O | 1.591455 | –1.492948 | –2.632511 |
| Fe | –1.893516 | –0.800300 | –0.613214 |
| C | 0.931392 | –0.233826 | 2.059671 |
| C | –0.518997 | –0.141879 | 2.878399 |
| C | –0.650019 | –1.218993 | 3.928116 |
| O | 1.282638 | 0.937189 | 1.544576 |
| O | –1.313872 | 0.695422 | 2.590496 |
| H | 0.625527 | –0.994609 | 1.318999 |
| H | 1.657639 | –0.606040 | 2.794173 |
| H | –0.068068 | –0.915631 | 4.805170 |
| H | –1.701646 | –1.324528 | 4.197461 |
| H | –0.246021 | –2.162272 | 3.552834 |
| O | –1.884445 | –1.826422 | 0.574199 |
| | $Fe_2(MoO_4)_3$+PPA | | |
| Mo | –2.651794 | 2.296464 | –0.653183 |
| Fe | 0.237873 | 1.702631 | 0.431373 |
| O | –2.161538 | 2.803355 | –2.217969 |
| O | –1.295103 | 2.623634 | 0.630583 |
| O | –4.086553 | 3.133888 | –0.239074 |
| O | –2.950523 | 0.367252 | –0.643071 |
| Mo | 2.198816 | 2.256402 | –2.257822 |
| O | 1.703889 | 3.626112 | –3.158549 |
| O | 3.912294 | 2.206148 | –2.292598 |
| O | 1.626680 | 2.392195 | –0.452059 |
| O | 1.445633 | 0.650526 | –3.006981 |
| Mo | 0.831738 | –1.075851 | –2.469855 |
| O | 1.098948 | –2.078822 | –3.825750 |
| O | –0.185609 | 0.433837 | –0.739317 |
| O | –1.041658 | –1.490104 | –2.029452 |
| O | 1.822916 | –1.690344 | –1.199327 |
| Fe | –1.566757 | –0.677303 | –0.591840 |
| C | –0.025237 | –0.376763 | 2.551637 |
| C | –0.036054 | –1.558110 | 1.582683 |
| C | 0.107647 | –2.887878 | 2.315610 |
| O | 0.074371 | 0.806427 | 2.221107 |
| O | –1.286811 | –1.551522 | 0.890102 |
| H | –0.122976 | –0.584631 | 3.629856 |
| H | 0.792177 | –1.421185 | 0.873810 |
| H | 0.132316 | –3.702769 | 1.589441 |
| H | 1.044117 | –2.906374 | 2.884691 |
| H | –0.730800 | –3.052218 | 2.998954 |
| | $Fe_2(MoO_4)_3$+O+PPA | | |
| Mo | –2.916566 | 1.176285 | –0.331708 |
| Fe | 0.140190 | 0.980784 | 0.151559 |
| O | –2.559127 | 1.731903 | –1.937173 |
| O | –1.459646 | 1.611491 | 0.773466 |
| O | –4.346065 | 1.907450 | 0.258840 |
| O | –3.130795 | –0.742324 | –0.502233 |
| Mo | 1.719678 | 3.049088 | –0.769966 |
| O | 1.273095 | 4.687814 | –0.529681 |
| O | 3.298253 | 3.011173 | –1.437101 |
| O | 1.554825 | 2.010331 | 0.798542 |
| O | 0.363016 | 1.919569 | –1.613292 |
| Mo | –0.314649 | 0.525545 | –2.991445 |
| O | –0.907280 | 1.489346 | –4.271917 |
| O | –0.486697 | –0.314479 | –0.980535 |

| | | | |
|---|---|---|---|
| O | –1.615334 | –1.034402 | –2.986489 |
| O | 1.133582 | –0.160112 | –3.580160 |
| Fe | –1.814486 | –1.500927 | –1.358626 |
| C | 0.615478 | –1.218182 | 3.809329 |
| C | 0.340810 | 0.000909 | 2.890103 |
| C | 0.179274 | 1.333065 | 3.536555 |
| O | 0.681704 | –2.340597 | 3.372549 |
| O | 0.296577 | –0.236542 | 1.680955 |
| H | 0.742138 | –0.982906 | 4.884594 |
| H | –0.224555 | –2.684460 | –0.287449 |
| H | –0.257066 | 2.064328 | 2.859611 |
| H | –0.421514 | 1.235769 | 4.448095 |
| H | 1.175270 | 1.681557 | 3.844558 |
| O | –1.068111 | –2.907475 | –0.736985 |
| $Fe_2(MoO_4)_3$+MeGO | | | |
| Mo | –2.505502 | 2.150071 | –1.076351 |
| Fe | 0.105110 | 1.594088 | 0.505421 |
| O | –1.876449 | 2.104635 | –2.674193 |
| O | –1.202983 | 2.722056 | 0.211959 |
| O | –3.856631 | 3.195237 | –1.048812 |
| O | –3.026364 | 0.366702 | –0.499084 |
| Mo | 2.234579 | 2.306322 | –1.850441 |
| O | 1.554782 | 3.675501 | –2.625361 |
| O | 3.940309 | 2.455163 | –1.907009 |
| O | 1.651978 | 2.199025 | –0.017957 |
| O | 1.623796 | 0.696348 | –2.638788 |
| Mo | 0.508790 | –0.874412 | –2.446093 |
| O | 0.235584 | –1.303587 | –4.077150 |
| O | –0.310877 | 0.319666 | –0.710042 |
| O | –1.315792 | –1.591896 | –1.784355 |
| O | 1.547248 | –2.070296 | –1.798570 |
| Fe | –1.736250 | –0.778755 | –0.379326 |
| C | 0.303623 | –0.365442 | 2.400757 |
| C | –0.337467 | –1.524920 | 1.976969 |
| C | 0.120000 | –2.880584 | 2.428510 |
| O | –0.102118 | 0.855752 | 2.113338 |
| O | –1.412281 | –1.524711 | 1.205301 |
| H | 1.165718 | –0.444338 | 3.063839 |
| H | 0.466134 | –3.458059 | 1.563350 |
| H | 0.930289 | –2.811619 | 3.156934 |
| H | –0.720226 | –3.424950 | 2.871157 |
| $Fe_2(MoO_4)_3$+O+MeGO | | | |
| Mo | –2.299561 | 1.742462 | 0.027715 |
| Fe | 0.777277 | 1.418993 | 0.031975 |
| O | –2.357121 | 1.781316 | –1.704371 |
| O | –0.547630 | 2.385641 | 0.585375 |
| O | –3.618706 | 2.591417 | 0.710010 |
| O | –2.201904 | –0.055816 | 0.539046 |
| Mo | 2.170012 | 2.886474 | –1.898632 |
| O | 2.077673 | 4.594187 | –1.882250 |
| O | 3.382177 | 2.432799 | –3.012665 |
| O | 2.383738 | 2.123578 | –0.124444 |
| O | 0.478664 | 1.974394 | –1.977583 |
| Mo | –0.330070 | 0.260509 | –2.890606 |
| O | –1.140454 | 0.853178 | –4.271781 |
| O | 0.351055 | –0.121600 | –0.645589 |
| O | –1.462260 | –1.062783 | –2.105170 |

| | | | |
|---|---|---|---|
| O | 1.054594 | –0.531495 | –3.511762 |
| Fe | –1.092342 | –1.202935 | –0.346188 |
| C | 0.796386 | 0.271905 | 2.826216 |
| C | –0.680162 | 0.360404 | 3.259458 |
| C | –1.370986 | –0.932010 | 3.588695 |
| O | 1.295360 | 0.696142 | 1.788677 |
| O | –1.130595 | 1.478466 | 3.404814 |
| H | 1.491632 | –0.173144 | 3.559824 |
| H | –2.429346 | –0.739693 | 3.770580 |
| H | –1.255382 | –1.664488 | 2.783496 |
| H | –0.918858 | –1.360478 | 4.493256 |
| O | –0.942191 | –2.605423 | 0.308904 |
| | | $Fe_2(MoO_4)_3$+HA | |
| Mo | –2.309050 | 1.034389 | –0.865463 |
| Fe | 0.718926 | 1.113256 | –0.348855 |
| O | –1.887107 | 1.122086 | –2.570022 |
| O | –0.866013 | 1.833630 | 0.093566 |
| O | –3.825947 | 1.755560 | –0.519351 |
| O | –2.233691 | –0.738453 | –0.320779 |
| Mo | 2.508437 | 2.493300 | –1.857949 |
| O | 2.525055 | 4.208441 | –1.901914 |
| O | 3.879563 | 1.922873 | –2.710099 |
| O | 2.357343 | 1.802753 | –0.055050 |
| O | 0.802491 | 1.715724 | –2.292156 |
| Mo | 0.060422 | –0.072116 | –3.381025 |
| O | –0.529804 | 0.357604 | –4.926364 |
| O | 0.424702 | –0.379608 | –1.085833 |
| O | –0.884604 | –1.594027 | –2.739488 |
| O | 1.653562 | –0.641133 | –3.698054 |
| Fe | –0.739225 | –1.691138 | –0.891074 |
| C | –0.158050 | –0.216860 | 2.319127 |
| C | –0.455102 | –1.709402 | 2.127091 |
| C | –0.715626 | –2.507097 | 3.370391 |
| O | 0.906602 | 0.219905 | 1.453401 |
| O | –0.512559 | –2.246699 | 1.018687 |
| H | 0.119207 | –0.025754 | 3.358305 |
| H | –1.065207 | 0.353455 | 2.090681 |
| H | –1.074560 | –3.505356 | 3.119601 |
| H | 0.208110 | –2.581342 | 3.958134 |
| H | –1.449872 | –1.990802 | 4.000691 |
| H | 1.605996 | 0.691812 | 1.940806 |
| | | $Fe_2(MoO_4)_3$+O+HA | |
| Mo | –2.322984 | 1.757731 | –0.665690 |
| Fe | 0.751707 | 0.893892 | –0.326943 |
| O | –2.396980 | 2.106582 | –2.351803 |
| O | –0.512437 | 2.044724 | –0.014704 |
| O | –3.484948 | 2.718157 | 0.146430 |
| O | –2.567365 | –0.087026 | –0.387434 |
| Mo | 2.211360 | 2.095977 | –2.410143 |
| O | 2.039842 | 3.788208 | –2.579278 |
| O | 3.451805 | 1.576870 | –3.466087 |
| O | 2.441461 | 1.551438 | –0.609660 |
| O | 0.547832 | 1.040647 | –2.429801 |
| Mo | 0.086106 | –0.609751 | –3.462638 |
| O | –0.127693 | –0.320465 | –5.132736 |
| O | 0.055532 | –0.660741 | –0.254499 |
| O | –1.461237 | –1.289322 | –2.680750 |

| | | | |
|---|---|---|---|
| O | 1.391653 | –1.710261 | –3.284762 |
| Fe | –1.444746 | –1.389108 | –0.878464 |
| C | 1.136455 | –0.405570 | 2.361035 |
| C | –0.328406 | –0.571264 | 2.781281 |
| C | –0.678766 | –1.927993 | 3.349305 |
| O | 1.296838 | 0.796676 | 1.568104 |
| O | –1.125117 | 0.334337 | 2.650442 |
| H | 1.480931 | –1.281225 | 1.802997 |
| H | 1.747570 | –0.289796 | 3.263611 |
| H | –1.586762 | –1.850586 | 3.949354 |
| H | –0.869360 | –2.615171 | 2.515555 |
| H | 0.135946 | –2.350409 | 3.947715 |
| O | –1.742344 | –2.767489 | –0.223496 |
| H | 2.232105 | 1.078937 | 1.563059 |
| | | $Fe_2(MoO_4)_3$+FD | |
| Mo | –2.051516 | 0.640811 | 1.303324 |
| Fe | 0.997665 | 0.220121 | 1.291314 |
| O | –1.915149 | 0.946690 | –0.423259 |
| O | –0.406023 | 1.131358 | 2.042645 |
| O | –3.336835 | 1.530576 | 1.996128 |
| O | –2.284599 | –1.251164 | 1.479486 |
| Mo | 2.766397 | 1.630498 | –0.278140 |
| O | 3.023172 | 3.316531 | –0.097270 |
| O | 3.926989 | 1.018592 | –1.378758 |
| O | 2.750393 | 0.710699 | 1.401267 |
| O | 0.914856 | 1.121014 | –0.569020 |
| Mo | –0.189459 | –0.214771 | –1.754202 |
| O | –0.881983 | 0.637323 | –3.057636 |
| O | 0.208598 | –1.061172 | 0.427741 |
| O | –1.355492 | –1.747465 | –1.320438 |
| O | 1.160240 | –1.003176 | –2.455506 |
| Fe | –1.186293 | –2.103438 | 0.382132 |
| C | 1.783905 | –0.337020 | 3.932494 |
| O | 1.180624 | –0.787157 | 2.961767 |
| H | 2.254034 | 0.654055 | 3.907065 |
| H | 1.853476 | –0.951906 | 4.839866 |

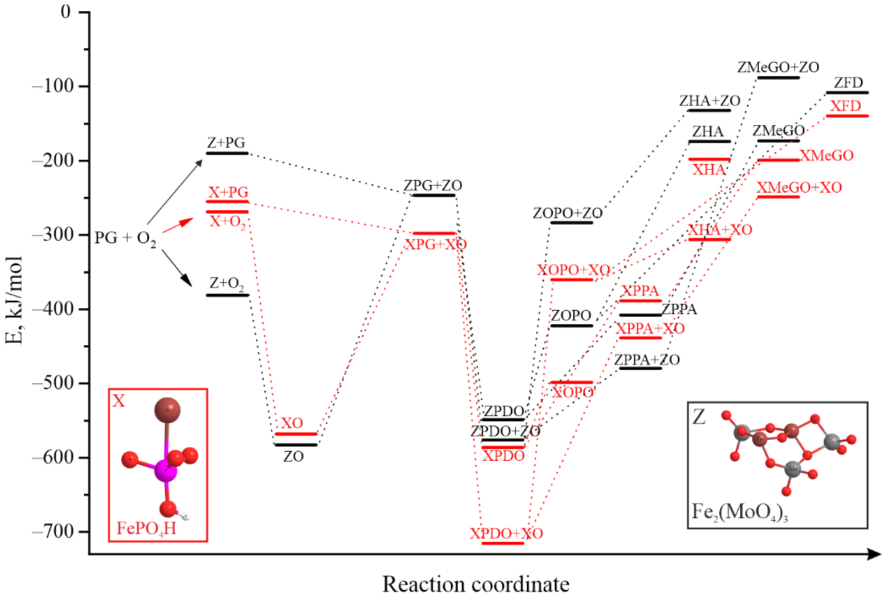


**Figure S4.** Interactions of key reagents, intermediates and products with active sites along the reaction coordinate (B3LYP/DGDZVP level of theory).
